# Reviewing the Rare Association Between Progestogen-only Implant and Pulmonary Embolism in a Woman with Multiple Cardiovascular Risk Factors: A Case Report

**DOI:** 10.7759/cureus.71728

**Published:** 2024-10-17

**Authors:** Adeyinka Owolabi, Rabiu Momoh

**Affiliations:** 1 Internal Medicine, Conquest Hospital, Hastings, GBR; 2 Critical Care, Medway Maritime Hospital, Gillingham, GBR

**Keywords:** contraception, multiple cardiovascular risk factors, progestogen-only implant, pulmonary embolism, women health

## Abstract

Pulmonary embolism (PE) is a recognized complication of hormonal contraceptive use, but the risk associated with progestogen-only implants in women with multiple cardiovascular (CV) risk factors remains uncertain. We present the case of a woman in her early forties with multiple CV risk factors, including obesity, obstructive sleep apnoea, and hypertension, who developed bilateral PE while using a progestogen-only implant. This case highlights the potential increased risk of PE in women with multiple CV risk factors using progestogen-only implants, warranting further investigation and cautious clinical decision-making.

## Introduction

Pulmonary embolism (PE) is a type of venous thromboembolism (VTE) in which dislodged thrombi from deep veins of the body occlude the pulmonary arterial system. It can be potentially life-threatening if not diagnosed and treated promptly, and accounts for most deaths from VTE [1].

Hormonal contraceptives have an established association with VTE, especially PE [2]. The various hormonal contraception options include combined hormonal contraception (CHC), progestogen-only contraception (POC), and intrauterine contraception (IUC). The POC types include progestogen-only implants, progestogen-only injectables, and progestogen-only pills [3]. Similarly, the existence of cardiovascular risk factors such as obesity, smoking, diabetes, hypertension, dyslipidaemia, and obstructive sleep apnea (OSA) are associated with increased risk of PE [4]. Of the hormonal contraceptives, only IUC, progestogen-only pills, and progestogen-only implants are recommended for use in those with multiple cardiovascular risk factors. However, there is limited available evidence (Class C recommendation) on the risk of VTE with the use of progestogen-only implants as a contraceptive option in this group of women [5]. The higher confidence level regarding the medication under review that can be attained with the prescribing healthcare provider and the end-user (patient group) regarding the likelihood of PE developing by virtue of this medication use can be negatively impacted if higher evidence-based recommendation levels cannot be demonstrated. 

The aim of this case report is to document the occurrence of PE in a female patient with multiple cardiovascular risk factors, on an etonogestrel implant (ENG-IMP), the only approved progestogen-only implant in the United Kingdom. We also hope to highlight the need for more robust evidence on the safety profile of progestogen-only implant use in women with multiple cardiovascular risk factors.

## Case presentation

A 41-year-old woman, fully active, with a body mass index (BMI) of 45.3 kg/m^2^, presented to the emergency unit on account of a two-day history of worsening shortness of breath, wheeze, non-specific chest discomfort, and occasional productive cough. She reported exertional breathlessness in the few months preceding the index admission, but no orthopnoea, paroxysmal nocturnal dyspnoea (PND), or body swelling was reported. She noted no fever or any other flu-like symptoms. The review of other systems was not significantly relevant.

She had a past medical history of OSA (and was on a regular continuous positive airway pressure (CPAP) device), hypertension, anxiety, and depression. She had a history of menorrhagia and had been on ENG-IMP for about four years for contraception. She had an 18-pack-year smoking history but quit about 11 years prior to this hospital presentation. She had no personal history of clotting or autoimmune disorders. Her regular medications included trazodone, sertraline, and felodipine. There was no known family history of clotting disorders. She had no recent long-distance travel history.

**Figure 1:**
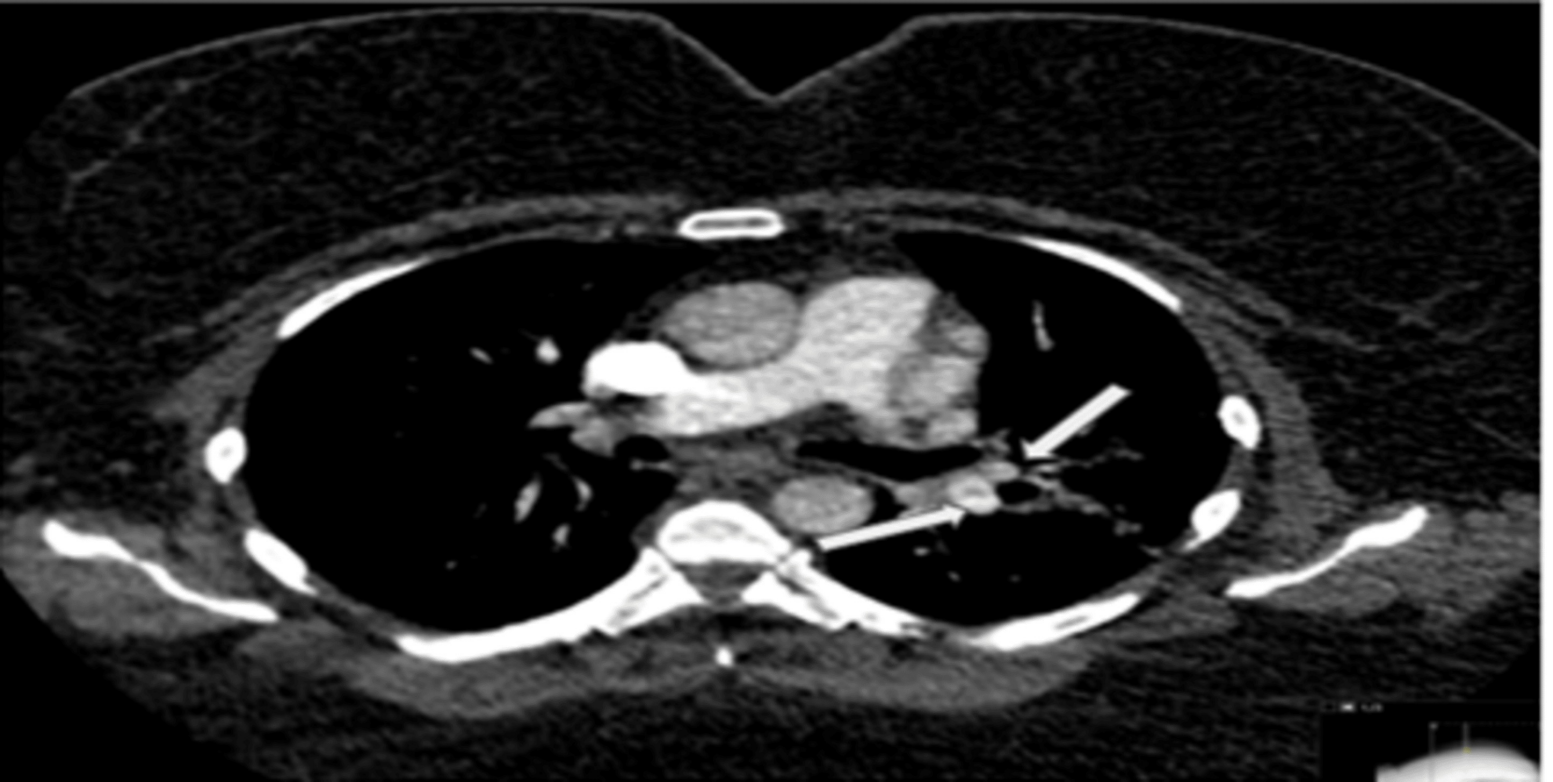
Axial CT pulmonary angiogram showing partial filling defects within the left lower lobe and lingular segment branches

**Figure 2:**
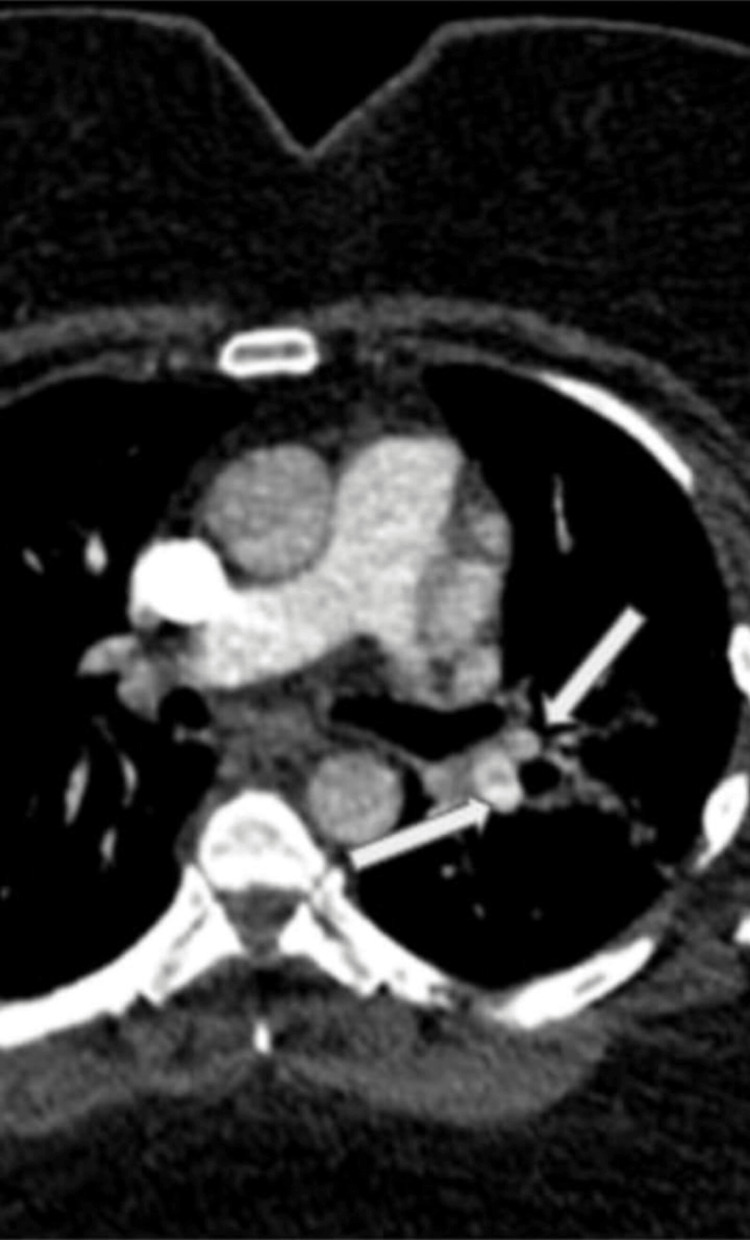
Zoomed-up axial CT pulmonary angiogram section showing partial filling defects within the left lower lobe and lingular segment branches

**Figure 3:**
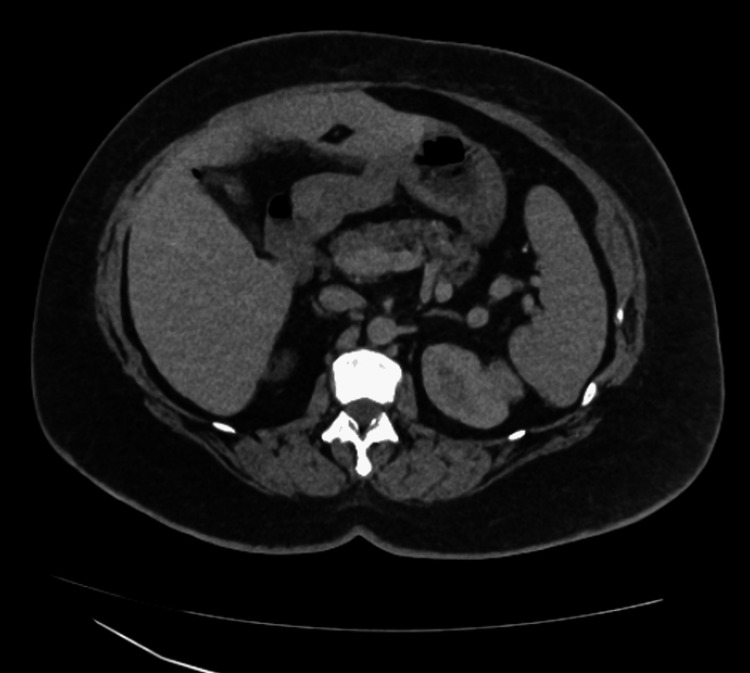
CT abdomen/pelvis (transverse section) showing no evidence of malignancy

**Figure 4:**
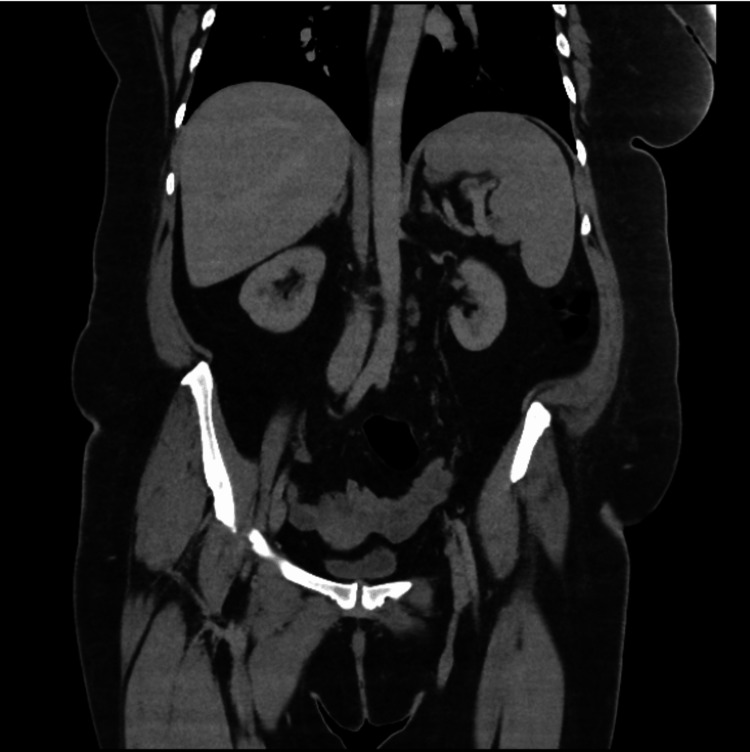
CT abdomen/pelvis (coronal section) revealing no evidence of malignancy

On initial examination at admission, she was afebrile, pale (+), anicteric, and hydrated, with no significantly enlarged peripheral lymph nodes, no leg swelling, and no calf swelling/tenderness or varicose veins. Her vital signs were as follows: temperature: 37.1 degrees Celsius, pulse rate: 103 beats per minute, blood pressure: 132/67 mmHg, respiratory rate: 23 cycles per minute, oxygen saturation: 95% (on room air). Chest examination revealed mild bilateral wheeze. The neurologic examination did not reveal any deficit. Cardiovascular system examination showed a regular fast pulse, no raised jugular venous pulsation with first and second heart sounds heard. Abdominal examination revealed truncal obesity, soft, not tender with normal bowel sound. She was initially commenced on treatment for a possible exacerbation of an undiagnosed chronic obstructive pulmonary disease. Her Wells score was low (1.5), but D-dimer was positive (481 ng/ml, lab reference: < 420 ng/ml) (Table 1) warranting further assessment with computed tomography pulmonary angiography (CTPA). CTPA confirmed bilateral PE, filling defects in the pulmonary arteries, predominantly peripheral on the right, but in the centripetal left lower lobe pulmonary artery and lingula segment of the left upper lobe and pulmonary infarction in the ligula segment (Figure 1, Figure 2). Her blood studies revealed the presence of iron deficiency anaemia (IDA) with haemoglobin 92 g/l (ref: 120-150 g/l), mean corpuscular volume 62.2 fL (ref: 80-100 fL), transferrin saturation 3% (ref: 15-50%) (Table 1). A computed tomography study of the abdomen and pelvis done as part of the assessment for IDA was negative for any underlying malignancy (Figure 3, Figure 4).

**Table 1:** Relevant deranged blood test results noted in the patient

Tests	Results	Reference ranges
Haemoglobin	92 g/l	120-150 g/l
Mean corpuscular volume	62.2 fL	80-100 fL
Transferrin saturation	3%	15-50%
D-dimer	481 ng/ml	< 420 ng/ml

The patient's PE Severity Index (PESI) score was 41 which put her at a class 1, very low mortality risk. She was started on treatment with a direct oral anticoagulant (DOAC), apixaban, and her case was reviewed with the haematology team who advised an initial six-month treatment with apixaban and for further review to decide if long-term anticoagulation will be indicated. She was on treatment in the hospital for three days and she was referred to the sexual health clinic on discharge where the ENG-IMP was switched to an intrauterine device (IUD).

## Discussion

This report highlights a case of a middle-aged lady with multiple cardiovascular risk factors who was on a progestogen-only implant, who presented to the hospital with an initial low clinical suspicion risk for PE (low-risk Wells score), but a positive D-dimer test warranted further assessment with CTPA, confirming a diagnosis of PE.

Anderson et al., in their review, using data obtained from a community-wide study that evaluated 1231 patients treated for VTE across 16 short-stay hospitals, categorised obesity as one of the weak risk factors for VTE/PE [6]. Similarly, the 2019 ESC guideline for the diagnosis and management of acute PE categorised obesity, hypertension and diabetes as weak risk factors for PE (odds ratio (OR) < 2), while fracture of lower limbs, hospitalisation for heart failure or atrial fibrillation/flutter, hip or knee replacement, major trauma, myocardial infarction, previous VTE and spinal cord injury were identified as strong risk factor (OR > 10) [7]. These risk factors, namely morbid obesity, OSA and hypertension, are regarded as cardiovascular risk factors, and just like arterial thrombosis, they have been found to be weakly associated with PE [6,7]. The cumulative effect of these multiple cardiovascular risk factors is an area that requires more exploration, even when the individual risk factors are considered ‘weak’ for VTE risk. To the best of our knowledge, no study has investigated the synergistic effect of these multiple cardiovascular risk factors on VTE and/or PE risk.

Regarding hormonal contraceptives in general, there is evidence to support an established association with VTE, especially PE [2]. The presence and strength of these associations vary across the different types of hormonal contraceptives with most of the evidence coming from the use of combined hormonal contraceptive methods. Several studies reported significantly increased risk of VTE with CHC methods, including combined oral contraceptives (COCs), transdermal patches and vaginal rings [8-11]. Conversely, most studies on the risk of VTE with the use of POC methods did not show any increased risk, except for progestogen-only injectables. Lidegaard et al., in their cohort study, did not find any increased risk of VTE with progestogen-only pills [8]. Similarly, the study by Hylckama et al. did not find an increased risk for VTE with the use of levonorgestrel-containing intrauterine device (IUD) [12]. However, Lawal et al. reported a case of PE in a 34-year-old woman, with no other identified risk factor, who had progestogen-only implant insertion two months prior [13].

Rosano et al., in their narrative review, provided compelling evidence on the synergistic impact of obesity and CHC on overall cardiovascular risk [11]. There is, however, a paucity of data on the association of progestogen-only implants with VTE risk, most especially in the setting of multiple cardiovascular risk factors where the association could be stronger. The grade 2 recommendation of progestogen-only implant use in individuals with multiple cardiovascular risk factors is from very limited available evidence [5,11]. The index patient under review had an ENG-IMP insertion in line with the United Kingdom Medical Eligibility Criteria for Contraceptive Use (UKMEC) provision but developed PE four years into the usage. With a potential synergistic effect of multiple cardiovascular risk factors in an individual, as in the present case, the risk of PE with progestogen-only implant (ENG-IMP) may be stronger than the present acknowledgement.

The title of this report accurately suggests that we aim to review and possibly raise the notion that the occurrence of VTE is possible with progestogen-only implant use as against definitively proving that there is a direct causality relationship between the two entities. The impact of the presence of other cardiovascular risk factors would be confounding factors in a move to absolutely prove the existence of a direct causality. More research is thereby suggested. Education between the prescriber and end-users (patients) can be better guided by virtue of this case report.

## Conclusions

We reported the case of a middle-aged woman with multiple cardiovascular risk factors who was using ENG-IMP for contraception and presented to the hospital with a diagnosis of PE. While there is currently a grade 2 recommendation for progestogen-only implants in the presence of multiple cardiovascular risk factors, clinicians should maintain a high index of suspicion for PE in patients with multiple cardiovascular risk factors using progestogen-only implants, especially when they present with unexplained breathlessness. Further studies are needed to explore the risk of PE associated with progestogen-only implants in women with multiple cardiovascular risk factors. As was the eventual situation in the patient under review, the role of alternative contraceptive methods such as IUDs for these high-risk individuals should be considered. This case highlights the importance of careful assessment and individualized decision-making in the choice of contraceptives for women at risk of PE.
